# Intensive behavioural interventions based on applied behaviour analysis (ABA) for young children with autism: A cost-effectiveness analysis

**DOI:** 10.1371/journal.pone.0270833

**Published:** 2022-08-16

**Authors:** Robert Hodgson, Mousumi Biswas, Stephen Palmer, David Marshall, Mark Rodgers, Lesley Stewart, Mark Simmonds, Dheeraj Rai, Ann Le Couteur

**Affiliations:** 1 Centre for Reviews and Dissemination, University of York, York, United Kingdom; 2 Bristol Myers Squibb, Lawrence Township, New Jersey, United States of America; 3 Centre for Health Economics, University of York, York, United Kingdom; 4 Centre for Academic Mental Health, School of Social and Community Medicine, University of Bristol, Bristol, United Kingdom; 5 Population Health Sciences Institute, Newcastle University, Newcastle upon Tyne, United Kingdom; National Center for Global Health and Medicine, JAPAN

## Abstract

**Background:**

The economic and social costs of autism are significant. This study evaluates the cost-effectiveness of early intensive Applied Behaviour Analysis (ABA)-based interventions for autistic pre-school children in the UK.

**Methods:**

A de novo economic analysis was developed in Microsoft Excel comparing early intensive ABA-based interventions compared with treatment as usual (TAU). The analysis used 15.5-year time horizon, with costs and benefits discounted a 3.5%. The model structure was based on cohort structure to capture changes in adaptive behaviour and cognitive ability over time. The analysis was informed by an individual patient data (IPD) meta-analysis of available evidence.

**Results:**

Adopting a public sector perspective, early intensive ABA-based therapies were associated with greater incremental costs and greater benefits. When pessimistic assumptions were made regarding the long-term effects of treatment incremental costs were £46,103 and incremental quality-adjusted life years (QALYs) were 0.24, resulting in an incremental cost-effectiveness ratio (ICER) of £189,122 per quality-adjusted life year (QALY). When optimistic assumptions were made about long-term effects, incremental costs were £39,233 with incremental benefits of 0.84 QALYs. The resulting ICER was £46,768 per QALY. Scenario analyses emphasised the importance of assumptions made regarding adult outcomes and type of school attended, both of which significantly affect the results of the analysis.

**Conclusions:**

The results of this economic analysis suggest that early intensive ABA-based interventions are unlikely to represent value for money, based on a £20,000 to £30,000 per QALY threshold typically adopted to inform UK healthcare funding decisions. However, important gaps in the available evidence, limit the strength of the conclusions that can be drawn from the presented analysis. Further research, focusing on the trajectory of autistic children following intervention is likely to be highly beneficial to resolving some of these uncertainties.

## Introduction

Autism spectrum disorder (henceforth referred to as autism) has significant social and economic impacts for individuals, their families and wider society [1–3]. Although the skills and needs of autistic children and their families are highly variable, they can have a profound effect on children’s development into adulthood [4, 5]. For example, the available evidence suggests that autistic adults are likely to have poorer educational attainment, lower rates of employment, are less likely to be living independently, and experience higher rates of mental and physical health problems compared with the general population and adults with other disabilities [6–8]. The estimated total costs to the United Kingdom (UK) of supporting autistic people and related conditions has been estimated at between £32.1 and £34.4 billion per year, with higher lifetime care costs reported for individuals with co-occurring intellectual disability [9].

Early intensive applied behaviour analysis (ABA) based interventions include a range of interventions that aim to positively impact a child’s developmental by shifting a child’s developmental trajectory through early interventions [10] and are typically delivered to young autistic children for several years on a one-to-one basis, for between 20 to 50 hours per week [11].

Early intensive ABA-based interventions have been variably defined to include a range of specific interventional approaches but minimally include early intensive behavioural interventions (EIBI) as first described by Lovaas in the late 1960s [10]. EIBI, as developed by Lovaas, is a behavioural interventional approach based on the principles of ABA and emphases teaching skills through structured task completion and rewards. EIBI has subsequently been adapted and developed to incorporate the principles of ABA within a more naturalistic and developmentally informed framework. Collectively known as Naturalistic Developmental Behavioural Interventions (NDBIs) [12], these techniques combine behavioural based techniques with child-led and incidental teaching. Specific examples include Pivotal Response Treatment (PRT) [13] and the Early Start Denver Model (ESDM) [14]. For the purposes of this study, we use ‘early intensive ABA-based interventions’ in the broad sense to refer to both EIBI and NDBI based approaches. Early intensive ABA-based interventions are not routinely delivered in the UK as part of state early years provision. There is, however, some provision for school-age children in parts of the country, as well as private provision for those able to self-fund [15–18]. Expanding early intensive ABA-based intervention provision would require significant investment on behalf of UK local authorities who are responsible for providing regional education and social care services. It may also require investment from clinical commissioner groups and local National Health Services (NHS) trusts who are responsible for regional health care expenditure. Several economic analyses have been conducted considering the value of implementing early intensive ABA-based interventions [19–21]. These studies suggest these interventions are highly cost-effective. However, none consider a UK perspective and they make several strong assumptions, particularly regarding effectiveness and long-term outcomes associated with early intensive ABA-based interventions. Most of the previous studies also only account for costs and do not consider the value of health improvements and other benefits.

The objective of this study was to evaluate the potential effectiveness and cost-effectiveness of early intensive ABA-based interventions in pre-school children from a UK perspective as part of a National Institute for Health Research (NIHR) funded health technology assessment (HTA). The full technical report [22] including an individual patient data (IPD) meta-analysis of the available primary research evidence on the effectiveness of early intensive ABA-based interventions is reported elsewhere [23]. This study presents the assessment of the cost-effectiveness of early intensive ABA-based intervention in a UK context. Notwithstanding the studies reported above, we are aware that cost-effectiveness analysis has been rarely conducted to assess interventions for treatment management of autism. For those unfamiliar with the underlying principles, we recommend readers consult one of the many introductory texts [24–26].

As part of the HTA, the economic evaluation utilised data from the IPD meta-analysis which evaluated the effectiveness of early intensive applied behaviour analysis (ABA)-based interventions for preschool autistic children compared with treatment as usual (TAU). Details of the IPD-meta-analysis including a justification of the adopted inclusion criteria and methods reported in full in the technical reports as well as in associated publication [22]. In brief, the IPD-meta-analysis found that early intensive ABA-based interventions produced statistically significant improvements in cognitive ability and adaptive behaviour after two years as compared to treatment as usual (including eclectic interventions), with limited evidence of effects on other outcomes. The studies that informed these estimates were, however, found to be at high risk of bias, with all studies using non-randomised designs and in many cases convenience samples. Further, while the nature of the interventions meant that blinding of education staff and participants was not possible, outcome assessors were also often not blinded to intervention. Importantly from the perspective of the economic analysis, most studies also lacked long-term follow-up data, meaning there was minimal evidence to support effects beyond two years.

## Methods

The economic analysis sought to compare early intensive ABA-based interventions with TAU which may include eclectic interventions in a population of pre-school autistic children. To assess and compare the cost-effectiveness of early intensive ABA-based interventions and TAU we considered both expected costs and utility (effectiveness) associated with them. Early intensive ABA-based interventions were assumed to represent any type of intensive ABA-based intervention and did not distinguish between subtypes (e.g. EIBI vs NDBIs). This was due to lack of evidence to support differential effects between subtypes [22]. TAU was modelled to represent current provision for young children with autism in the UK.

The de novo analysis was designed and developed in collaboration with UK and international experts and was also able to draw on an advisory group that included representation from autistic people, parents and practitioners (see S1 File). To reflect the fact that autism is a long-term neurodevelopmental condition and is likely to accrue benefits and have cost implications that extend beyond the healthcare system, the economic analysis was undertaken from both health and social care services, and a public sector perspective which included costs accruing to the education sector. The health and social care services perspective included all health benefits along with costs incurred by the health sector, such as any direct NHS medical costs and costs of social care. The public sector perspective added a broader range of costs borne by the state, including, for example, costs of providing education.

Expected utilities were expressed in quality-adjusted life years (QALYs) which are a function of quality and length of life [27], to allow for a direct comparison with other health care interventions and for incremental cost-effectiveness ratio (ICER; additional quality-adjusted life years (QALY) divided by additional costs) to be estimated. The advantage of this approach is that it allows the result of the economic analysis to benchmarked against willingness to pay (WTP) criteria commonly used to inform decisions on the value of healthcare technologies in the UK. To benchmark the potential cost-effectiveness of early intensive ABA-based interventions, ICERs were compared with the UK National Institute for Health and Care Excellence (NICE) published decision rules, which outline a WTP threshold used in the NHS (£20,000 to £30,000 per QALY) and set the maximum ICER acceptable for new expenditure in the NHS [28]. We, however, note that alternative willingness to pay thresholds may be relevant to implementation decision in this context because at least some of the funding and benefits of autism interventions lie outside the health sector. There are, however, no set WTP for other sectors as cost-utility analysis is not routinely used in other sectors e.g. education. Costs and outcomes were discounted using a 3.5% annual discount rate, in line with current NICE guidelines [28].

### Model structure

**Fig 1:**
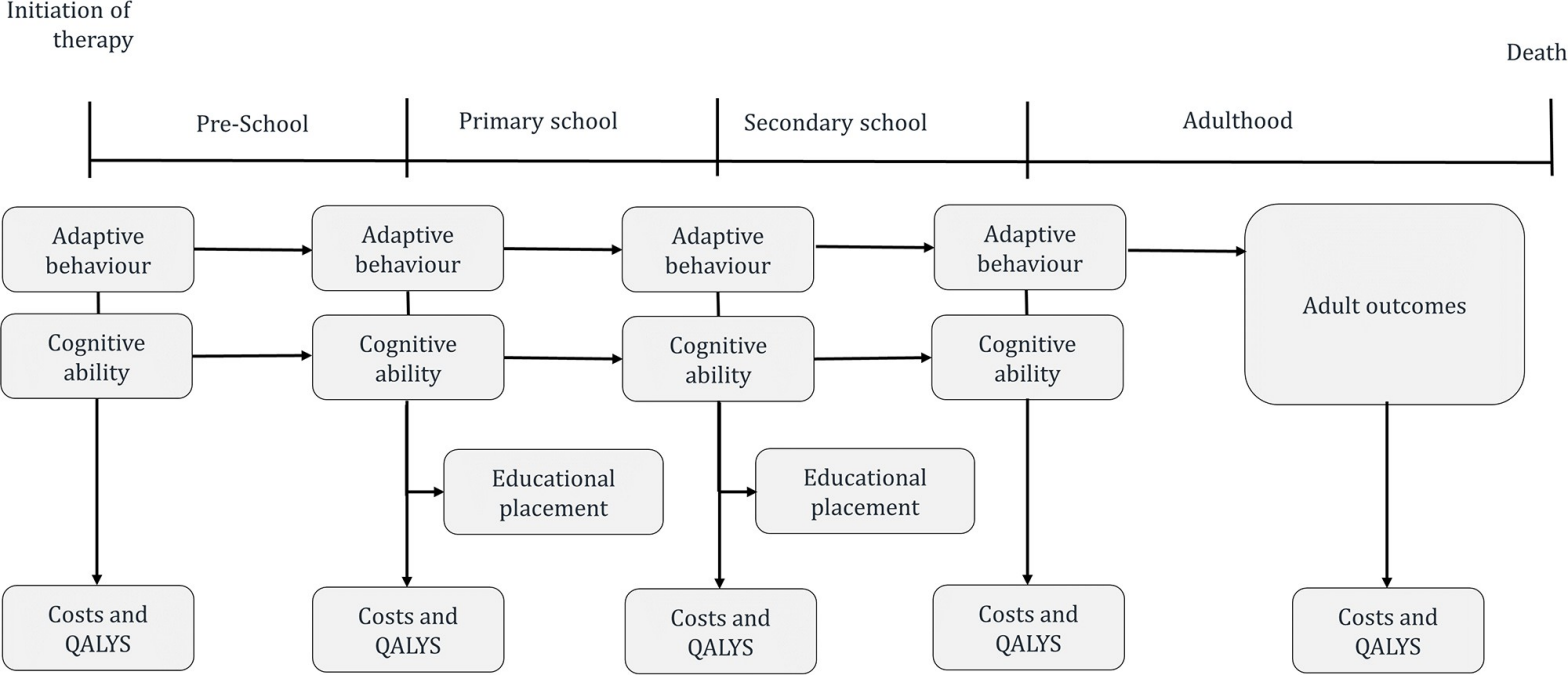
Model structure.

The economic analysis was constructed in Microsoft Excel^®^ and used a simple cohort approach. Under this approach mean cognitive ability scores (IQ) and Vineland Adaptive Behaviour scores (VABs) for the cohort are used to predict model outcomes [29]. These included educational costs, social care and medical costs as well as quality of life measured in QALYs. The focus on cognitive ability and adaptative behaviour reflects the limitations of the available effectiveness which primarily reports on changes in these two outcome measures [30]. An overview of the model structure is described in Fig 1. The analysis uses a cycle length of one month (representing the shortest time period over which outcomes were measured) with a half-cycle correction applied to correct for the fact that events can occur at any point in a cycle. Changes in mean cognitive ability and adaptative behaviour scores were therefore incorporated as mean changes per month.

The time horizon considered in the analysis was assumed to be 15.5 years, to represent the period from age 3 years to the average age children leave secondary education (18.5 years). The relatively short time horizon of 15.5 years was selected because of considerable uncertainty about the long-term effects of early intensive ABA-based interventions and the lack of epidemiological evidence linking short and long-term outcomes [23].

The time horizon in the main analysis was divided into three phases representing different stages of an individual’s childhood. The first phase “pre-school” (up to 4.5 years old) reflects the period when early interventions are delivered, while the second “school-age” is split into two sub-phases covering the period in which children attend primary (4.5 to 11.5 years old) and secondary education (11.5 to 18.5 years old). To accommodate educational placement and the associated resource implications, the analysis distinguished between three education settings: mainstream, mainstream with support, and special education provision. Estimation of the proportion of children receiving each type of education was determined at the beginning of the primary and secondary phase of the model and estimated using logistic regression models. These determined the proportion of children in each educational setting based on the cognitive ability and adaptative behaviour scores of the cohort, with separate models fitted for primary and secondary age children, see Table 1 for the coefficients applied and data sources.

**Table 1:** Parameter inputs used in the economic analysis.

**Baseline characteristics of children**						
	**Mean**	**SE**	**Distribution**	**Lower bound**	**Upper bound**	**Source (references)**
**Proportion male**	87.57%	1.47%	Beta-bionomial	84.69%	90.45%	IPD Meta-analysis
**Proportion with intellectual disability (IQ<70)**	82.95%	3.32%	Beta-bionomial	57.32%	61.55%	IPD Meta-analysis
**Age in months**	36	0	NA	NA	NA	Assumption
**Adaptive behaviour (VABS)**	63.19	0.43	Beta-bionomial	62.35	64.04	IPD Meta-analysis
**Cognitive ability (IQ)**	59.43	1.08	Beta-bionomial	57.32	61.55	IPD Meta-analysis
**Autism symptom severity (ADOS)**	6.98	0.18	Beta-bionomial	6.63	7.33	IPD Meta-analysis
**Natural history**						
	**Mean**	**SE**	**Distribution**	**Lower bound**	**Upper bound**	**Source**
**Adaptive behaviour (VABs)** *	—0.45	1.27	Normal	-2.94	2.04	[31–34]
**Cognitive ability** *	-0.28	1.24	Normal	-2.70	2.14	
**Short-term treatment effect year 1**						
	**Mean**	**SE**	**Distribution**	**Lower bound**	**Upper bound**	**Source**
**Adaptive behaviour (VABs)** *	2.92	2.46	Normal	-1.90	7.76	IPD Meta-analysis
**Cognitive ability (IQ)** *	9.16	2.44	Normal	4.38	13.93	
**Short-term treatment effect year 2**						
	**Mean**	**SE**	**Distribution**	**Lower bound**	**Upper bound**	**Source**
**Adaptive behaviour(VABs)** *	7.00	2.58	Normal	1.95	12.06	IPD Meta-analysis
**Cognitive ability (IQ)** *	14.13	2.54	Normal	9.16	19.10	
**Education type regression coefficients Primary school**						
	**Coefficient**	**SE**	**Distribution**	**Lower bound**	**Upper bound**	**Source**
**Adaptive behaviour (VABs)**	-0.08	0.02	Normal	-0.12	-0.042	[34, 35]
**Cognitive ability (IQ)**	-.02	0.01	Normal	-0.051	0.006	
**Cut 1 (mainstream with support)**	-8.75	0.02	Normal	-11.08	-6.42	
**Cut 2 (specialist schooling)**	-6.10	0.86	Normal	-7.781	-4.41	
**Education type regression coefficients Secondary school**						
	**Coefficient**	**SE**	**Distribution**	**Lower bound**	**Upper bound**	**Source**
**Adaptive behaviour (VABs)**	-0.04	0.02	Normal	-12.48	-6.27	[36]
**Cognitive ability (IQ)**	-0.06	0.02	Normal	-9.32	-3.89	
**Cut 1 (mainstream with support)**	-9.37	1.58	Normal	-0.084	-0.001	
**Cut 2 (specialist schooling)**	-6.60	1.39	Normal	-0.089	-0.025	
**Educational placement based (observed values applied in scenario analysis only)**						
	**Coefficient**	**SE**	**Distribution**	**Lower bound**	**Upper bound**	**Source**
**Mainstream (ABA)**	30%	N/A	N/A	N/A	N/A	[10, 34, 35]
**Mainstream with support (ABA)**	38%	N/A	N/A	N/A	N/A	
**Special education (ABA)**	32%	N/A	N/A	N/A	N/A	
**Mainstream (TAU)**	1%	N/A	N/A	N/A	N/A	
**Mainstream with support (TAU)**	27%	N/A	N/A	N/A	N/A	
**Special education (TAU)**	72%	N/A	N/A	N/A	N/A	
**Regression model used to predict quality of life in Children**						
	**Coefficient**	**SE**	**Distribution**	**Lower bound**	**Upper bound**	**Source**
**Constant**	−0.2438	0.2015	Beta-bionomial	-0.639	0.154	[37]
**Age**	0.0119	0.0186	Beta-bionomial	-0.025	0.048	
**Age2**	0.0003	0.0010	Beta-bionomial	-0.002	0.001	
**ADOS score**	-0.0063	0.0078	Beta-bionomial	0.0071	0.013	
**Cognitive ability (IQ; Log)**	0.0304	0.0478	Beta-bionomial	-0.063	0.124	
**Adaptive behaviour (VABs)**	0.0103	0.0016	Beta-bionomial	-0.021	0.009	
**Costs of interventions**						
	**Mean**	**SE**	**Distribution**	**Lower bound**	**Upper bound**	**Source**
**ABA-based interventions**	£36,682.78	£7,336	Gamma	£22,303	£51,062	Micro-costed [38, 39]
**TAU**	£8,634.33	£1,726	Gamma	£5,249	£12,019	Assumption/personnel communication.
**Social care and medical costs**						
	**Coefficient**	**SE**	**Distribution**	**Lower bound**	**Upper bound**	**Source**
**Intercept**	£1,900.09	£762.41	Normal	$405.	£3394	[36]
**Adaptive behaviour (VABs)**	-£8.78	£13.98	Normal	-£36.18	£18.63	
**Cognitive ability (IQ)**	-£7.81	£10.99	Normal	-£29.35	£13.74	
**Costs of Schooling**						
	**Mean**	**SE**	**Distribution**	**Lower bound**	**Upper bound**	**Source**
**Mainstream School**	£4,417.70	£883	Gamma	£2,686	£6,149	[36]
**Supported**	£8,689.78	£1,737	Gamma	£5,283	£12,096	
**Special School**	£15,702.78	£3,140	Gamma	£9,547	£21,858	
**Adult care costs**						
	**Mean**	**SE**	**Distribution**	**Lower bound**	**Upper bound**	**Source**
**Own home/parents**	£0.00	NA	NA	NA	NA	[38, 40]
**Sheltered -Low intensity**	£53,274.88	£10,654.98	Gamma	£32,391	£74,159	
**Sheltered—High intensity**	£99,336.44	£19,867.29	Gamma	£60,397	£13,8276	
**Residential**	£115,553.00	£23,110.60	Gamma	£70,256	£16,0850	
**Day services**	£17,728.57	£3,545.71	Gamma	£10,779	£24,678	
**Respite care**	£1,927.00	£385.40	Gamma	£1,172	£2,682	
**Employment support**	£290.00	£58.00	Gamma	£176	£404	
**Adult education**	£4,159.00	£831.80	Gamma	£2529	5789.328	
**Hospital**	£43.00	£8.60	Gamma	£26.14	59.86	
**Other health and social services**	£726.00	£145.20	Gamma	£441	£1,010	

ABA = Applied Behaviour Analysis, ADOS = Autism Diagnostic Observation Schedule, IQ = intelligence quotient, SE = Standard error, TAU = Treatment as Usual, VABs = Vineland Adaptive Behavior Scales

* Annual changes in scores

### Inputs

To populate the inputs used in the economic analysis we conducted a series of formal systematic reviews and undertook additional primary data analysis to develop and populate the economic analysis. Details of each review and data sources are provided in the full technical report [22]. An overview of the structure of the economic analysis and input parameters is outlined below. A summary table of all input parameters used in the analysis are reported in Table 1.

Uncertainty in parameter inputs was explored in deterministic sensitivity and scenario analysis as well as in probabilistic sensitivity analysis (PSA) which represent input parameters as distributions around the mean estimate. The PSA was undertaken using Monte Carlo sampling methods, using 10,000 samples. The choice of distribution for individual parameters was selected in accordance with their statistical suitability, see Table 1.

Starting characteristics except age were drawn from the IPD meta-analysis, these included biological sex and the outcome measures: Autism Diagnostic Observation Schedule (ADOS) [41], cognitive ability (IQ) and adaptive behaviour (VABS). Starting age was set to 3 to ensure consistency and differed only slightly from the average baseline age (3.16 years) reported by studies included in the IPD meta-analysis.

#### Treatment effect

In the TAU arm of the economic analysis, changes in cognitive ability and adaptive behaviour scores were modelled using the four studies identified in the systematic review that had follow-up duration greater than two years [31–34]; these were then interpolated assuming a linear trend. These predicted small declines in cognitive and adaptive behaviour measures and aligned with much of the epidemiological research which show small declines in scores over time [42–48].

To model the treatment effect, in the early intensive ABA-based arm, cognitive ability and adaptive behaviour scores were modelled by applying the treatment effect derived from the IPD meta-analysis [23]. Cognitive ability and adaptative behaviour scores used in the early intensive ABA-based interventions arm of the economic analysis were therefore the sum of the score predicted for TAU plus the treatment effect for intensive ABA.

The treatment effect was modelled in two phases: a short-term phase, covering the first two years and a long-term phase, covering two years and onwards. This distinction was made to reflect that most of the studies included in the IPD meta-analysis had no more than two years of follow-up. In the first two years (up to cycle 24), the economic analysis applied the treatment effect at one year and two years from the IPD meta-analysis (see Table 1 for input values). Evidence on the longer-term effects of early intensive ABA-based interventions on cognitive ability and adaptive behaviour scores, and in particular the degree to which any early benefits are sustained, is very limited. Very few studies identified in the systematic review reporting outcomes post end of therapy and there have been relatively few attempts to follow up children after conclusion of the short-term studies, with a few notable exceptions [49–51]. To reflect this uncertainty in long-term effects of treatment both optimistic and a pessimistic long-term scenarios were explored.

Under the optimistic scenario, the treatment effect at 2 years was assumed to persist throughout the time horizon of the analysis, while under the pessimistic scenario it was assumed that the treatment effect dissipated over time, such that at seven years no additional treatment effect with ABA-based interventions remained. This time limit reflects the maximum period of follow- up by a study included in the IPD meta-analysis [34] acknowledging that any further changes in the treatment effect are unknown. In both scenarios, these increases/decreases were modelled as a linear trend because evidence from intermediate time points was unavailable.

### Mortality

Several epidemiological studies have demonstrated that people with autism experience reduced life expectancy relative to the general population. To account for this, autism specific mortality rates were applied using relative risks reported in Hirvikoski et al. [52] and applied to general population rates reported by the UK Office of National Statistics. Mortality was, however, not linked to intervention effectiveness.

### Health-related quality of life

A systematic search of studies reporting utility scores of autistic children was undertaken and identified several studies- full details of this review are reported in the full technical [22]. Of the studies identified, only one reported utility scores in a way that could be linked meaningfully with the outcomes reported in the IPD meta-analysis (this was primarily because reported values did not differentiate between different levels of severity/ability). This study recruited 224 children (aged 4–17 years) with autism and related conditions, including children with co-existing intellectual disability [37]. Quality of life was estimated at each time point based on reported algorithms, using adaptive behaviour, age, baseline cognitive ability (IQ) and baseline autism symptom severity (ADOS scores) as predictors of quality of life. Quality of life scores therefore changed in accordance with changes in cognitive ability, adaptive behaviour scores and age over time. In the lifetime horizon scenario analysis, the age parameter was held constant at 18 years and age-related decrements applied to account for the natural effects of ageing on utility scores. These decrements were calculated based on published vales using a published algorithm of utility scores in autistic children [53].

### Resource use

The base price year was 2016/17 as this was the most recent year of publication for Personal Social Services Research Unit (PSSRU) [38] available and inflation indices for 2017/2018 were not available at the time. Prices reported in alternative cost years were inflated using inflation indices reported in PSSRU.

For the purposes of this study, early intensive ABA-based intervention was defined as consisting of 30 hours of one-to-one sessions [23]. The duration of treatment was set at 24 months as this aligns with the maximum follow-up period available for the majority of the clinical research studies included in the IPD meta-analysis [23]. It is, however, acknowledged that in practice children may continue to receive ABA-based treatment, including top-up therapy, through the school years. The modelled assumptions may therefore underestimate the true costs of ABA-based interventions. The economic analysis also does not account for discontinuation rates as no study reported compliance of fidelity to task.

Costs for early intensive ABA-based interventions were derived from published sources [38, 39] and set at £36,682 per annum. Costs of TAU were primarily based on UK national funding structures and information obtained from three local authorities on special educational needs (SEN) funding. The estimated cost of nursery provision was £8,634 per annum. The analysis also considered local authority provision of TAU intervention with costs based on funding information provided by the City of York and Newcastle local authorities [54].

UK costs associated with each type of school education and the costs for social and medical (NHS) care were drawn from a re-analysis of IPD obtained from Barrett et al [36] in a sample of adolescents with autism and Asperger’s syndrome. These costs were then linked to outcomes in the economic analysis using a regression equation in which cognitive ability (IQ) and adaptive behaviour scores (VABs) were used to predict total social care and medical costs. UK care costs in adulthood were based primarily on values reported in Buescher et al. [9] and values reported in PSSRU [38].

### Sensitivity and scenario analysis

Uncertainty in parameter inputs was explored in deterministic sensitivity and scenario analysis as well as in probabilistic sensitivity analysis. Details of which are explained below.

#### Deterministic sensitivity analysis

One-way deterministic sensitivity analysis was performed to explore the impact of single parameters on the results of the economic analysis. In this analysis each parameter was varied according to its 95% CI or standard error, while holding all other parameters constant. All parameters with uncertainty were included in the sensitivity analyses, this excluded time horizon and discount rates, which were assumed to be fixed. For a detailed list of the parameters varied and range of variation tested in the one-way univariate sensitivity analysis, see Table 1.

#### Scenario analysis

Two scenario analysis were explored key assumptions made the in the base-case economic analysis.

The first explored the data used assess the impact of each intervention on education outcomes. In the base-case analysis education outcomes are modelled using an indirect link in which cognitive ability and adaptive behaviour scores are used to predict education outcomes. This approach allows for a consistency in the studies used to inform the relative effectiveness of early ABA-based interventions. Several studies, however, report direct evidence on the impact of early ABA-based interventions and therefore can be used to directly inform education outcomes avoiding the need for an indirect link. Scenario analysis therefore explores using these studies to inform education placement. In this scenario analysis, children are assumed to attend the same type of education throughout childhood (see Table 1 for proportions used).

The second scenario analysis explored a lifetime time horizon. This acknowledges that early interventions may have long-lasting and potentially permanent effects on an individual that last not only through childhood but into adulthood. In this scenario an additional phase was added to the structure of the economic analysis, with five levels of independence: “Completely independent”, “Mostly independent”, “Some independence”, “Mostly dependent” and “Completely dependent”. The definitions used for each category were based on those used in Howlin et al., [4] see technical report for details [22].

Independence levels were determined on entering the adult phase of the analysis based on adaptive behaviour scores at 18.5 years of age. Due to the lack of available data regarding changes in independence over time, it was assumed that independence levels remained constant throughout adulthood. The validity of this assumption is uncertain, as it does not include any consideration of an individual’s potential for change, or the impact of any existing or additional health and/or mental health needs.

Evidence linking adult levels of independence to childhood cognitive ability and adaptive behaviour is extremely limited. Although several studies report strong correlations between adult cognitive ability, adaptive behaviour scores and adult levels of independence, data are rarely reported in a way that allows predictions about adult outcomes to be made [47, 55–57]. Farley et al., [56] however, reports mean adaptive behaviour scores for several different independence levels. Using this summary, we were able to create simulated IPD to which a logistic regression model with independence levels as coefficients was fitted. This algorithm was then used to predict adult outcomes. The details of the methodology used are reported in the full technical report [22]. The coefficients generated from this analysis are reported in Table 1.

#### Probabilistic sensitivity analysis

Uncertainty in parameter inputs was also explored in a probabilistic sensitivity analysis which represents input parameters as distributions around the mean estimate. Distributions for each parameter input were informed by the 95% CI, and additional literature. The probabilistic analysis ran the analysis using10,000 samples and was undertaken using simple Monte Carlo sampling methods (this involves random draws from the distributions for each iteration of the economic analysis). The choice of distribution for individual parameters was selected in accordance with their statistical suitability, see Table 1. The probability that early intensive ABA-based interventions are cost-effective was also investigated by considering the proportion of iterations where early the ICER was below the willingness to pay threshold.

## Results

### Deterministic analysis

Table 2 presents the base-case results of the economic analysis using both a health and social care services payer perspective and a wider public sector perspective. In all scenarios, in line with the IPD-meta-analysis results [22], the analysis predicted that the use of early intensive ABA-based interventions was associated with improved outcomes. The magnitude of accrued benefits differed substantially depending upon the assumptions made about the durability of the treatment effect, with optimistic scenarios predicting QALY benefits more than three times greater than under the pessimistic scenarios. While incremental costs were always positive due to the implementation costs of early intensive ABA-based interventions, their magnitude differed substantially across scenarios. This was driven by the degree of cost offsets (due to lower medical or educational costs), resulting from improved outcomes, and was a function of the perspective taken. This was a consequence of the limited scope for costs offsets when adopting a health and social care services payer perspective and as such incremental costs are lowest when the broader public sector is adopted.

**Table 2:** Base-case results (deterministic analysis).

Scenario	Therapy	Costs	QALYs	Inc. cost	Inc. QALYs	ICER
**NHS and social services perspective**						
**Pessimistic**	ABA	£76,622	4.61	£57,879	0.24	£236,837
	TAU	£18,743	4.37	-	-	-
**Optimistic**	ABA	£75,976	5.21	£57,233	0.84	£68,362
	TAU	£18,743	4.37	-	-	-
**Public sector perspective**						
**Pessimistic**	ABA	£195,310	4.61	£43,940	0.24	£179,799
	TAU	£151,370	4.37	-	-	-
**Optimistic**	ABA	£187,612	5.21	£36,242	0.84	£43,289
	TAU	£151,370	4.37	-	-	-

ABA = Applied Behaviour Analysis, ICER = incremental cost-effectiveness ratio, Inc. = Incremental, QALY = Quality Adjusted Life Years, TAU = Treatment as Usual.

Using the UK NICE decision rules to benchmark the results of the cost-effectiveness analysis, early intensive ABA-based interventions would not be considered cost-effective in either the optimistic or pessimistic scenario. Under a health and social care services payer perspective, the pessimistic scenario suggests that early intensive ABA-based interventions would need to generate a further 1.68 QALYs or £50,547 in additional cost savings (not captured by the economic analysis) to meet the maximum NICE threshold of £30,000 per QALY. In the optimistic scenario, early intensive ABA-based interventions would only be cost-effective at a threshold of £30,000 per QALY if there were a further 1.07 QALYs or £32,117 in additional cost savings not captured by the analysis.

When adopting a public sector perspective, the size of the additional QALY benefits or cost savings required is lower. In the pessimistic scenario, early intensive ABA-based interventions would need to generate either a further 1.22 QALYs worth of additional health or non-health benefits or a further £36,608 in additional costs savings to be cost-effective at this threshold. In the optimistic scenario, this is reduced to either 0.37 QALYs worth of benefits or £11,126 in cost savings.

### Sensitivity analysis

**Fig 2:**
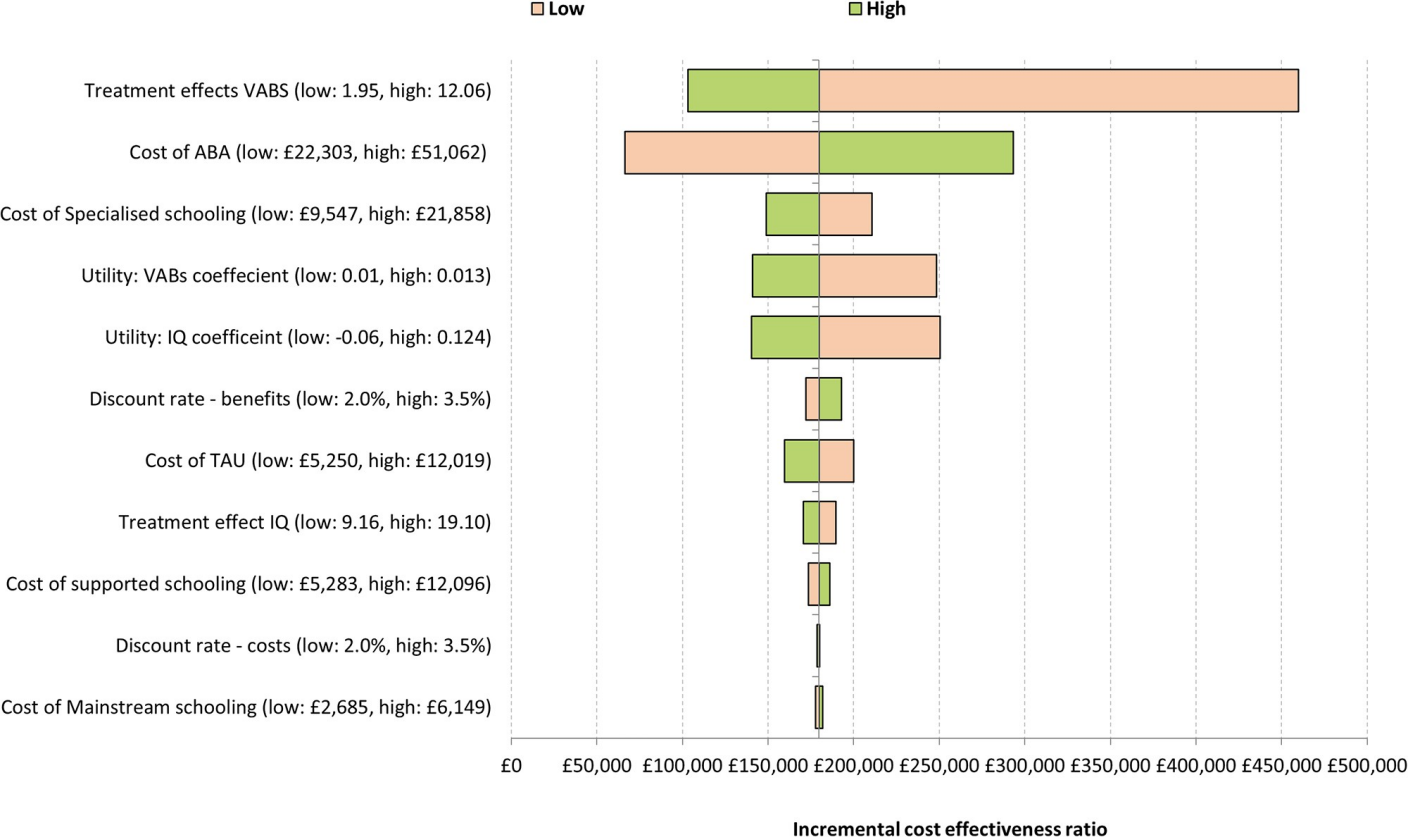
Tornado diagram (pessimistic analysis).

**Fig 3:**
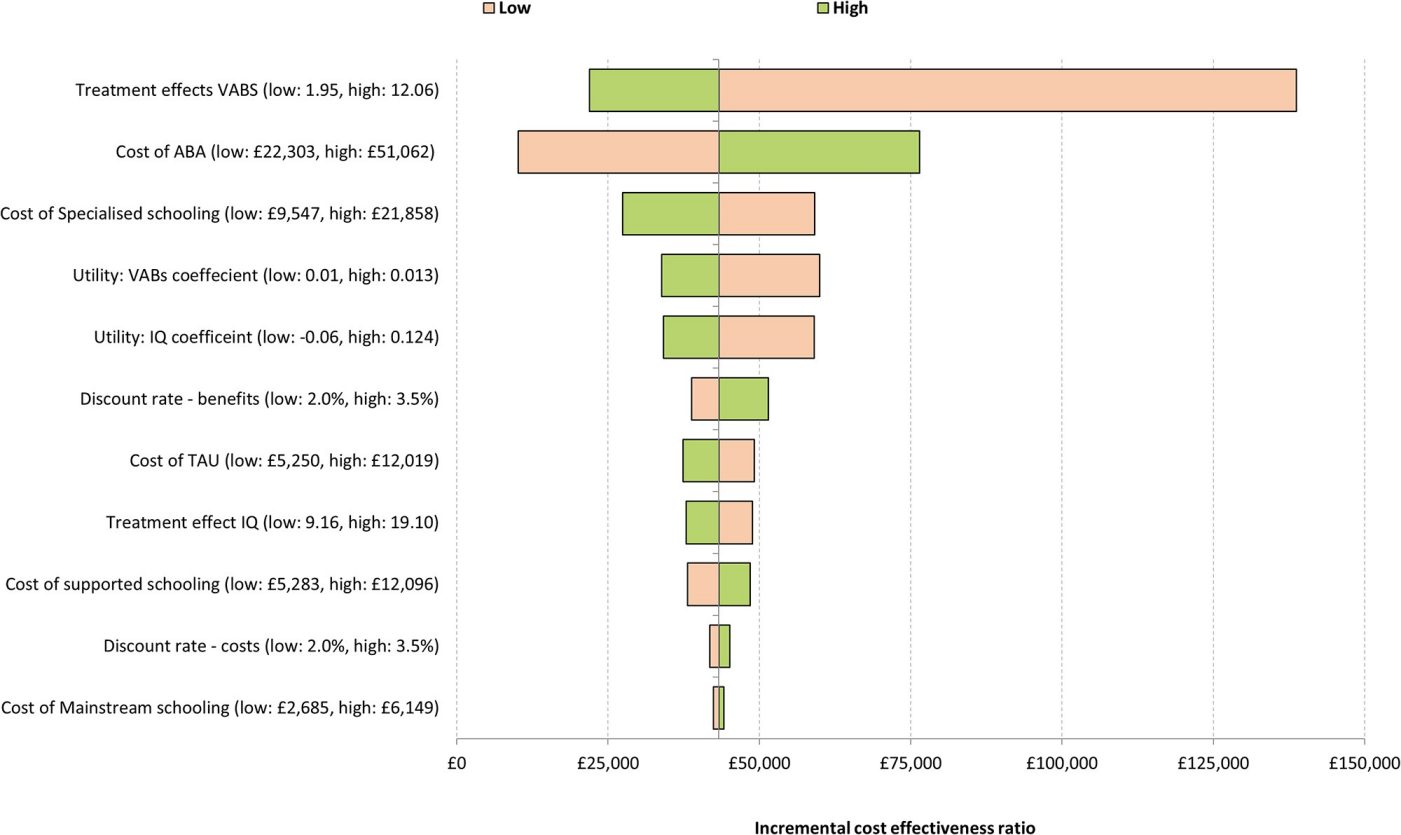
Tornado diagram (optimistic analysis).

Figs 2 and 3 present the results of the deterministic sensitivity analysis in the form of a tornado diagram. This summarises the results of the 10 most influential parameters on the ICER, with each bar representing the variation in the ICER for that parameter. These analyses indicate early intensive ABA-based interventions remained cost-ineffective across a large range of parameter values. The magnitude of the treatment effect and cost of specialised education were the key drivers of the analysis.

### Scenario analysis

Results of two scenario analysis are presented in Table 3 assuming a public sector perspective.

**Table 3:** Scenario analysis results: Public sector perspective.

Scenario	Therapy	Costs	QALYs	Inc. cost	Inc. QALYs	ICER
**Scenario 1: Adult outcomes**						
**Pessimistic**	ABA	£1,800,040	6.87	£43,940	0.24	£179,799
	TAU	£1,756,100	6.62	-	-	-
**Optimistic**	ABA	£1,705,806	8.49	-£50,294	1.86	Dominant*
	TAU	£1,756,100	6.62	-	-	-
**Scenario 2: Educational outcomes**						
**Pessimistic**	ABA	£170,113	4.61	£12,325	0.24	£50,435
	TAU	£157,788	4.37	-	-	-
**Optimistic**	ABA	£169,467	5.21	£11,680	0.84	£13,951
	TAU	£157,788	4.37	-	-	-

ABA = Applied Behaviour Analysis, ICER = incremental cost-effectiveness ratio, Inc. = Incremental, QALY = Quality Adjusted Life Years, TAU = Treatment as Usual.

*Dominant implies greater benefits at lower costs.

The first scenario assumes a lifetime horizon in which adult outcomes are included. The second replaces the regression analysis used to predict education placement with observed data on education outcomes from three studies included in the systematic review and IPD meta-analysis. [10, 34, 58].

The impact of adopting a longer time horizon depends upon assumptions made about the durability of the treatment effect. Under the pessimistic scenario, where the treatment effect dissipates to zero after seven years, the ICER does not differ from the base-case analysis. In contrast, in the optimistic scenario, early intensive ABA-based interventions were found to dominate TAU, now generating greater benefits and lower incremental costs. This is because the longer time horizon allows greater scope benefits to be accrued as well as resulting in additional cost-savings.

The impact of using the direct evidence on education outcomes is significant in both the pessimistic and optimistic scenarios, in both cases, the resulting ICER is substantially lower than in the base-case analysis as a consequence of reduced incremental costs. The ICER in the pessimistic scenario, however, remains above the NICE threshold of £30,000 per QALY gained.

### Probabilistic sensitivity analysis

The results of the probabilistic analysis are presented in Table 4 and broadly correspond with those of the deterministic analysis (see Table 2).

**Table 4:** Base-case results: Probabilistic analysis.

Scenario	Therapy	Costs	QALYs	Inc. cost	Inc. QALYs	ICER
**NHS and social services perspective**						
**Pessimistic**	ABA	£76,587	5.02	£58,940	0.24	£240,868
	TAU	£17,648	4.77	-	-	-
**Optimistic**	ABA	£76,341	5.60	£58,630	0.85	£69,385
	TAU	£17,711	4.75	-	-	-
**Public sector perspective**						
**Pessimistic**	ABA	£191,264	5.00	£46,103	0.24	£189,122
	TAU	£145,161	4.75	-	-	-
**Optimistic**	ABA	£184,411	5.61	£39,233	0.84	£46,768
	TAU	£145,178	4.77	-	-	-

ABA = Applied Behaviour Analysis, ICER = incremental cost-effectiveness ratio, Inc. = Incremental, QALY = Quality Adjusted Life Years, TAU = Treatment as Usual.

**Fig 4:**
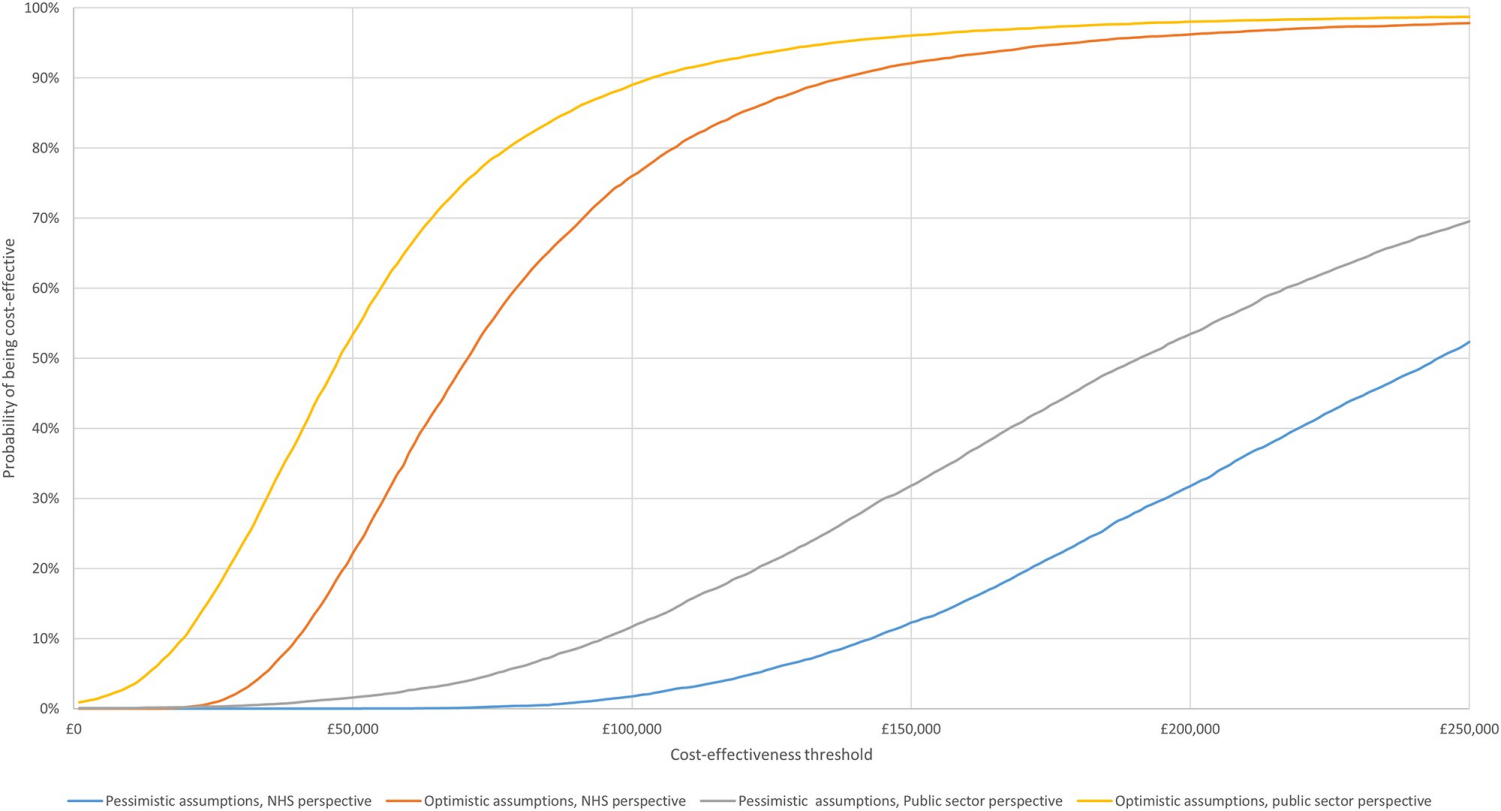
Cost-effectiveness acceptability curve (CEAC).

The degree of decision uncertainty around these estimates is illustrated in Fig 4. This presents the cost-effectiveness acceptability curve (CEAC), which describes the probability that early intensive ABA-based interventions are cost-effective at different WTP thresholds [59]. The probability that early intensive ABA-based interventions are cost-effective when taking a health and social care services payer perspective, remains close to zero up to a threshold of £84,000 per QALY in the pessimistic scenario and up to £25,000 per QALY in the optimistic scenario. When a public sector perspective is adopted, the probability that early intensive ABA-based interventions are cost-effective begins to depart from zero at a threshold of around £30,000 per QALY under the pessimistic scenario and £1,000 per QALY in the optimistic scenario.

## Discussion

This study presents the first assessment of the cost-effectiveness of early intensive ABA-based intervention from UK payer perspective, using data from a recently completed IPD meta-analysis [23].

Results indicated that early intensive ABA-based intervention might generate higher incremental QALYs than TAU intervention. Under both optimistic and pessimistic scenarios, the magnitude of these benefits is relatively small, given the additional costs of implementing early intensive ABA-based therapies. Interpretation of whether these benefits represent value for money is dependent on the willingness to pay threshold considered. In our analysis we have considered a £20,000 to £30, 000 threshold, which are commonly employed by NICE in the context of UK healthcare decisions. However, it is important to acknowledge that these thresholds do not necessarily apply to autism, owing to the associated impacts upon multiple sectors and therefore may not be appropriate for decision making. These decision rules do, however, provide a useful benchmark, indicating that even when adopting a public sector perspective, early intensive ABA-based interventions would not meet the decision criteria for implementation in the UK. This is irrespective of the assumptions made about the about the long-term effectiveness of early intensive ABA-based interventions.

The results of our analyses contrast sharply with those reported in previous economic evaluations which have overwhelmingly concluded that early intensive ABA interventions are highly cost-effective generating substantial cost savings [19–21]. The difference in our results is driven in large part by differences in assumptions made regarding the persistence of any treatment effect and the time horizon. Previous economic evaluations have tended to use longer life horizons and to assume that the benefits of early intensive ABA-based treatments persist for the lifetime of the analysis. These differences highlight the importance of these assumptions and align with the scenario analysis we present which shows that the cost-effectiveness of early intensive ABA-based interventions is likely contingent upon treatment effects persisting into adulthood.

In interpreting the results of our analyses, it is also important to acknowledge that there are likely to be substantive logistical hurdles to implementing early intensive ABA-based interventions in the UK, which may incur additional costs not accounted for in the current analysis. These include the challenges with recruiting and (re)training the very large number of therapists that would be required to implement this intervention across the UK, a difficulty aggravated by both the intensity of the intervention and the fact that the intervention is delivered on either a one to one or small group basis. Diagnosis of children within an appropriate time frame also represents a significant challenge. Although in some areas access to interventions is determined by a needs-based assessment rather than a full ASD diagnostic evaluation assessment, implementing wide scale provision of an early ASD specific intervention would likely require a significant expansion of diagnostic services. Current diagnostic patterns would mean that a substantial number of autistic children would not have access to treatment due to not being diagnosed until later childhood [60]. These challenges while probably not insurmountable would need significant irrevocable investment in services. This should be considered carefully when evaluating the risks and benefits of implementing early intensive ABA-based interventions, particularly in light of the significant uncertainties in the supporting effectiveness evidence.

### Limitations

The limitations of this analysis reflect the limitations of the underpinning research evidence. The narrow focus on cognitive ability and adaptive behaviour in existing effectiveness studies mean that the economic analysis cannot account for effects on other outcomes and consequently may not fully account for the benefits (or harms) of early intensive ABA-based interventions. The IPD meta-analysis also identified important weaknesses in the available research evidence [22]. Specifically, it raised concerns about the reliability of treatment effect estimates, with substantive weaknesses in the methods used; all studies were considered to be at high risk of bias. This has important implications for interpreting the results of the economic analyses if the benefits of early ABA-based interventions have not been clearly established and therefore it is not clear whether any quality of life benefits would actually be realised in practice. Further this analysis was also not able to consider relevant subgroups of children, as no substantive evidence of differential effects was established in the IPD meta-analysis.

Importantly, in common with most early intervention evaluation studies, there is little reliable longer-term follow-up data from children who have received early intensive ABA-based intervention to inform assumptions about the durability of initial effect and whether any comparative benefits of therapy relative to TAU are retained through childhood and even into adulthood. This is further compounded by limited epidemiological evidence on the prognostic value of the outcomes typically collected in the effectiveness evidence (cognitive ability and adaptive behaviour) [61–63] and a general lack of evidence about how (and if) therapy can alter the course of a child’s education, and impact on adult outcomes [35, 49] As demonstrated in the scenario analysis, considering longer term effects, resolving the uncertainties associated with extrapolating outcomes is crucial to more accurately assessing the cost-effectiveness of early interventions including intensive ABA-based interventions. Moreover, there is a need to better define what constitutes a good outcome for autistic adults and we note that recent literature has highlighted that functional outcomes such as employment status and independent living are not well correlated with the subjective well-being of individuals [64, 65]. The simplistic approach adopted in the presented scenario analysis may therefore poorly reflect the benefits of early intervention and the priorities of the autism community.

More generally, there is uncertainty regarding several input parameters as well as omissions that may impact on the validity of the analysis. For example, the HRQoL, data used were sourced from a study recruiting children who had less severe autism symptoms and included fewer children with cognitively impairments. These difference in the population may be important as the goals and ambitions relevant to children with more severe impairments may be quite different to a more able population and, as such, the way improvements in outcomes are valued may differ substantially. We were also not able to include (due to lack of appropriate data) HRQoL improvements that may accrue to carers. This may be an important omission in the context of early autism interventions. There is sizable literature demonstrating that parents of autistic children have lower HRQoL and significant potential for positive spillover effects [66–68].

Costing data on interventions is also likely to be subject to a high degree of uncertainty. Costings were based broadly on the early intensive ABA-based interventions considered in the IPD meta-analysis and what constitutes current TAU in the UK. There are, however, several factors that are likely to mean that these costings may not fully reflect the actual costs of provision. These include significant variations in the way in which early ABA-based interventions can be delivered (e.g. greater use of group settings), as well as significant variations in what constitutes current TAU in the UK.

### Suggested research priorities

The substantive uncertainties associated with evaluating the cost-effectiveness of early intensive ABA-based interventions may in part be resolved by new high-quality research studies that address the concerns identified in the IPD meta-analysis regarding the internal validity of the identified effectiveness evidence. Specifically, such studies should make use of pre-specified intervention evaluation protocols including an RCT design and blinded outcome assessment.

For future intervention evaluation trials, careful consideration and decisions about the choice of relevant outcomes for the autism community together with the use of reliable outcome measures will need to be prioritised. The autism community experts, researchers and practitioners consulted as part of the advisory group for this NIHR funded study highlighted the limitations of cognitive ability and adaptive behaviour to capture both benefits and any potential adverse impacts of the intervention. Concern was also expressed about the relevance of these outcomes for the autism community and for best practice. The development of a set of core outcome measures relevant for children with autism/ASD under the age of five years and their families would be extremely beneficial in this regard and would also facilitate the sharing of findings across clinical trials [63].

As highlighted above, little is known about the timeframes over which both benefits and harms from early intervention may become apparent. The length of follow-up of any future studies of early intervention in autism needs to be considered carefully as there is substantial uncertainty regarding the durability or otherwise of early benefits. Ideally, the longest possible follow-up is desirable, but recognising both financial and pragmatic constraints, other types of research may be needed to address these uncertainties. This could include, retrospective observations studies, or planned follow-up of children recruited to existing cohort studies.

## Conclusions

Based on the existing evidence, the results of this economic analysis indicate that early intensive ABA-based interventions are unlikely to represent value for money, using the current thresholds typically adopted by NICE to inform UK healthcare funding decisions. The presented economic analysis, however, highlights substantive uncertainties in several key inputs and assumptions which are likely to impact significantly on the cost-effectiveness of early intensive ABA-based interventions making it impossible to draw definite conclusions from the present study.

Future studies addressing these key uncertainties including high-quality comparative research studies of the short and longer-term impact of evidence-based early interventions may allow for more nuanced and robust estimates of the cost-effectiveness of such interventions for young autistic children.

## Supporting information

S1 File Advisory group membership. (DOCX)
