# Supplementary material for: Intensive behavioural interventions based on applied behaviour analysis (ABA) for young children with autism: A cost-effectiveness analysis
Source: PLoS One. 2022 Aug 16;17(8):e0270833. doi: 10.1371/journal.pone.0270833 (PMC9380934; doi:10.1371/journal.pone.0270833)
Supplement: S1 File — (DOCX) [file pone.0270833.s001.docx]

# S1. Advisory group membership

| **Name** | **Role** | **Organisation** |
| --- | --- | --- |
| Mike Clarke | Academic advisor (IPD and Statistics) | Queen’s University Belfast |
| Bernard Fleming | NAS Representative | National Autistic Society |
| Patricia Howlin | Academic advisory (Autism research) | Institute of Psychiatry |
| Emma Jenner | Parent | n/a |
| Helen McConachie | Academic advisory (Autism research) | Newcastle University |
| Anne McLaren | Parent | n/a |
| Dean McMillan | Clinical Academic | University of York |
| Damian Milton | Representative with ASD/ NAS Representative | National Autistic Society |
| Roo Philip | ABA practitioner | Tailor Ed Foundation |
| Tristram Smith | Clinical Academic and expert in ABA therapy | University of Rochester Medical Center |
| Peter Szatmari | Academic advisory (Autism research) | McMaster University |
| Emma Truelove | Education Psychologist | York City Council |
| Colin Wilson | Representative with ASD | n/a |
| Anne-Marie Wood | Parent | n/a |
| Suzy Yardley | ABA practitioner | Child Autism UK |
